# Prognostic implications of intratumoral CD103^+^ tumor-infiltrating lymphocytes in pulmonary squamous cell carcinoma

**DOI:** 10.18632/oncotarget.14632

**Published:** 2017-01-10

**Authors:** Jaemoon Koh, Sehui Kim, Moon-Young Kim, Heounjeong Go, Yoon Kyung Jeon, Doo Hyun Chung

**Affiliations:** ^1^ Department of Pathology, Seoul National University Hospital, Seoul National University College of Medicine, Seoul, Republic of Korea; ^2^ Department of Biomedical Sciences, Seoul National University College of Medicine, Seoul, Republic of Korea; ^3^ Department of Forensic Medicine, Seoul National University College of Medicine, Seoul, Republic of Korea; ^4^ Department of Pathology, Asan Medical Center, University of Ulsan College of Medicine, Seoul, Republic of Korea; ^5^ Tumor Immunity Medical Research Center, Tumor Microenvironment Global Core Research Center, Cancer Research Center, Seoul National University College of Medicine, Seoul, Republic of Korea; ^6^ Ischemic/Hypoxia Institute, Seoul National University College of Medicine, Seoul, Republic of Korea

**Keywords:** CD103, tumor-infiltrating lymphocytes, pulmonary squamous cell carcinoma, prognosis, cancer immunotherapy

## Abstract

CD103 is the α_E_ subunit of α_E_β_7_ integrin that is expressed in tissue-resident memory T cells, where it promotes cytotoxic T cell responses against tumors. However, little is known about its expression or clinicopathological implications in non-small cell lung cancer (NSCLC). This study investigated the prognostic implications of CD103^+^ tumor-infiltrating lymphocytes (TILs) in NSCLC. We established two cohorts: patients with resected NSCLC (*n =* 132) and patients with pulmonary squamous cell carcinoma (pSCC), a subset of NSCLC (*n =* 378), to estimate the prognostic significance of CD103^+^ TILs. The numbers of CD103^+^ TILs in the intratumoral (i.e., intraepithelial) and stromal regions of NSCLC were estimated using immunohistochemistry and automated image analysis. In the NSCLC cohort, high numbers of intratumoral CD103^+^ TILs were significantly associated with prolonged disease-free survival (DFS) and overall survival (OS) in patients with pSCC but not in those with pulmonary adenocarcinoma. In the pSCC cohort, a positive correlation was observed between the numbers of intratumoral CD103^+^ and CD8^+^ TILs (correlation coefficient = 0.736, *P* < 0.001). The ratio of intratumoral/stromal CD103^+^ TILs was higher in pSCC with high compared to low E-cadherin expression (*P* = 0.021). According to Kaplan-Meier analysis, high intratumoral but not stromal CD103^+^ TILs were associated with prolonged DFS and OS in patients with resected pSCC (*P* = 0.021 and 0.002, respectively). Multivariate analysis revealed that a high number of intratumoral CD103^+^ TILs is an independent predictor of a more favorable DFS (*P* = 0.021). Thus, a high number of intratumoral CD103^+^ TILs is a favorable prognostic indicator in patients with pSCC.

## INTRODUCTION

In recent decades, therapies targeting driver gene mutations in tumors have significantly improved the survival of patients with non-small cell lung cancer (NSCLC) [1]. Among the different types of NSCLC, druggable genetic changes have been variably observed in pulmonary adenocarcinoma (pADC), whereas these mutations are rarely found in pulmonary squamous cell carcinoma (pSCC). Moreover, acquired resistance to targeted therapy eventually develops via diverse mechanisms in almost all treated patients [2–4]. Thus, novel therapeutic strategies are needed to overcome these limitations in NSCLC.

Tumor surveillance involves dynamic interactions between tumor cells and variable cell components in the microenvironment, which are also targeted by cancer control strategies [5]. The tumor microenvironment is composed of heterogeneous cells including immune cells, endothelial cells, and fibroblasts. The immune system is critical for regulating tumor development and progression by balancing pro-inflammatory and anti-inflammatory responses [6]. Recently, several immune checkpoints such as the programmed cell death (PD)-1/PD-ligand 1 (PD-L1) and CTLA4 pathways have emerged as immunotherapy targets for solid tumors, particularly NSCLC, thereby improving patient survival [7]. Thus, a comprehensive understanding of immune cell network interactions in the tumor microenvironment is important in clinical oncology.

CD8^+^ cytotoxic T lymphocytes are critical effector cells in adaptive immunity involved in killing tumor cells [8]. Moreover, many studies have demonstrated that high numbers of CD8^+^ tumor-infiltrating lymphocytes (TILs) are significantly correlated with prolonged survival in patients with various cancers, including bladder, ovarian, and lung cancers [9–12]. Furthermore, interactions between tumor and immune cells via cell adhesion molecules are critical for maintaining immune responses against tumor cells [13]. Among the adhesion molecules, CD103 is the α_E_ subunit of the heterodimeric α_E_β_7_ integrin that mediates cell adhesion, migration, and signaling via interaction with its ligand, E-cadherin, expressed in epithelial cells [14]. Under homeostasis, CD103^+^ expression in memory T cells residing in peripheral tissues, including the skin and mucosa, increases the migration and retention of these cells [15, 16]. Moreover, the interaction between CD103 and E-cadherin is responsible for the recruitment and retention of antigen-specific TILs in gliomas, and ovarian cancer, and lung cancer [17–19]. CD103 expression was observed more frequently in the TILs of intratumoral (i.e., intraepithelial) than stromal regions, and this has been implicated in patient survival [20–22].

Recently, high numbers of CD103^+^ TILs were correlated with improved survival in patients with early stage NSCLC [20]. However, their prognostic implications in patients with NSCLC according to histological subtype and localization remain unclear. Thus, we investigated the expression pattern of CD103 in TILs according to the clinicopathological characteristics of patients with NSCLC.

## RESULTS

### Immunohistochemical analysis of intratumoral CD103^+^ TILs in NSCLC

**Figure 1:**
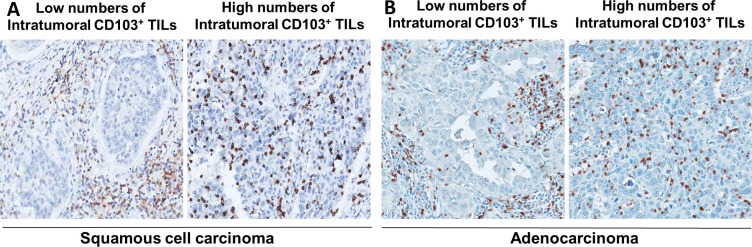
Representative immunohistochemical images of low versus high CD103^+^ cell numbers in intratumoral regions of pulmonary squamous cell carcinoma (**A**) and adenocarcinoma (**B**)

To investigate the potential prognostic value of intratumoral CD103^+^ TILs in NSCLC, we performed immunohistochemistry (IHC) analysis of CD103 in the NSCLC cohort (*n* = 132). The mean numbers of intratumoral and stromal CD103^+^ TILs/mm^2^ were 357.5 ± 368.9 and 225.0 ± 205.5 in patients with pSCC (*n* = 47) and 182.0 ± 220.9 and 233.0 ± 269.2 in patients with pADC (*n* = 85), respectively (Figure 1A and 1B). The correlation between the intratumoral CD103^+^ TIL number and the clinicopathologic features of patients with NSCLC are summarized according to histological subtype in Supplementary Table 1. High numbers of CD103^+^ TILs were correlated with male sex (*P* = 0.051) and smoking status (*P* = 0.023) in patients with ADC. Smokers exhibited higher numbers of intratumoral CD103^+^ TILs compared to non-smokers among the patients with pSCC; however, this difference was statistically insignificant, potentially due to the skewed population of non-smokers (*n* = 2) with pSCC. Kaplan-Meier survival analysis revealed a longer disease-free survival (DFS) (*P* = 0.038) and overall survival (OS) (*P* = 0.038) among patients with high intratumoral CD103^+^ TIL numbers in pSCC (Supplementary Figure 1A). However, the number of intratumoral CD103^+^ TILs was not significantly related to DFS or OS in patients with pADC (Supplementary Figure 1B).

### Comparative analysis of CD103^+^ and CD8^+^ TIL numbers in pSCCs according to clinicopathologic features

**Figure 2:**
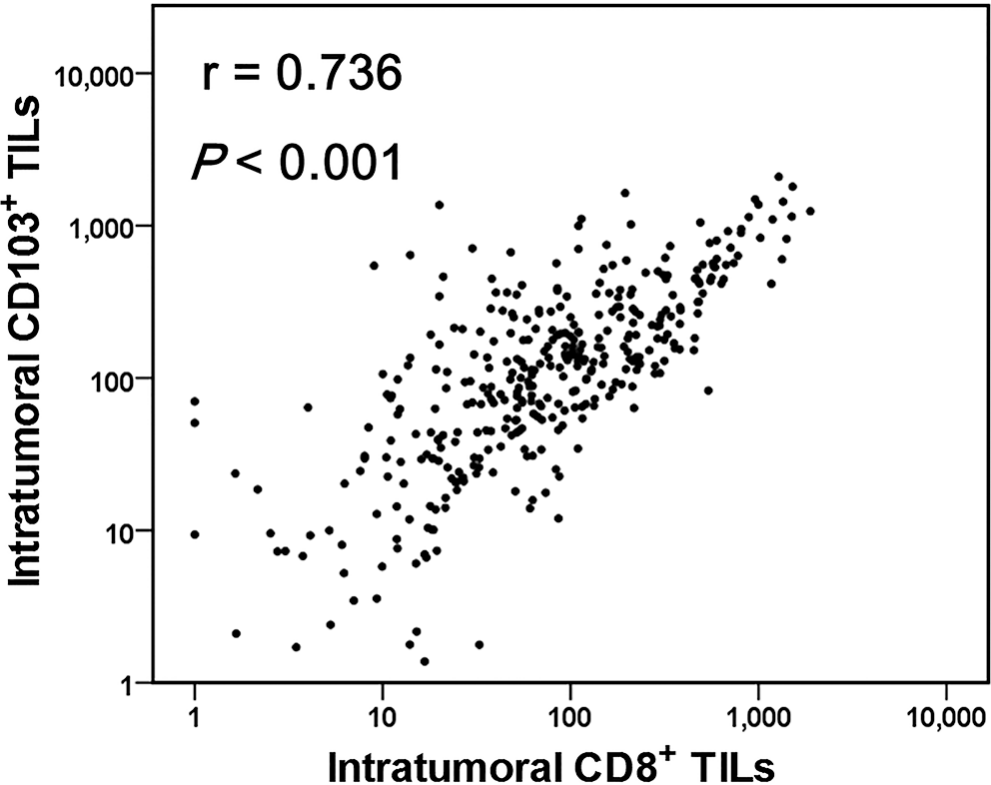
A strong positive correlation between the numbers of CD103^+^ and CD8^+^ tumor-infiltrating lymphocytes (TILs) in pulmonary squamous cell carcinomas (*n* = 378) Linear regression analysis was performed to determine the relationship between CD103^+^ and CD8^+^ TIL numbers, and the correlation was analyzed using Spearman's rank correlation coefficient.

Based on the CD103^+^ TIL results in the NSCLC cohort, we postulated that CD103^+^ TILs are significantly correlated with survival of patients with pSCC. To address this further, we established a cohort comprising a large number of patients with pSCC (*n* = 378). Several recent lines of evidence suggest that CD8^+^CD103^+^ TILs account for the majority of CD103^+^ TILs [18, 20, 23]. Thus, we analyzed CD8 and CD103 expression in TILs of pSCC intratumoral and stromal regions. In the intratumoral regions, the number of CD103^+^ TILs was higher than that of CD8^+^ TILs (222.2 ± 299.0 and 175.0 ± 268.8, respectively; *P* <0.001), whereas in the stromal regions, the number of CD8^+^ TILs was much higher than that of CD103^+^ TILs in stromal areas (173.2 ± 175.4 and 108.7 ± 185.0, respectively; *P <* 0.001). However, the numbers of CD103^+^ and CD8^+^ TILs were positively correlated in intratumoral regions (r = 0.736, *P* < 0.001, as shown in Figure 2). Moreover, the clinicopathological characteristics of patients with pSCC were evaluated with respect to the numbers of CD103^+^ and CD8^+^ TILs in intratumoral and stromal regions (Table 1). Tumor size was inversely correlated with the number of intratumoral CD103^+^ TILs (*P* = 0.037), whereas smokers exhibited significantly high numbers of stromal CD103^+^ (*P* < 0.001) and CD8^+^ TILs (*P* = 0.001) in the pSCC cohort.

**Table 1:** The numbers of intratumoral and stromal CD8^+^ and CD103^+^ tumor-infiltrating lymphocytes (TILs) according to patient clinicopathological characteristics in pulmonary squamous cell carcinoma cohort (n = 378)

Squamous cell carcinoma		Intratumoral CD103^+^ TILs			Stromal CD103^+^ TILs			Intratumoral CD8^+^ TILs		Stromal CD8^+^ TILs	
Clinicopathological characteristics		*n*	Mean ± SD (number/mm^2^)	*P*	Mean ± SD (number/mm^2^)	*P*	Mean ± SD (number/mm^2^)	*P*	Mean ± SD (number/mm^2^)	*P*
Sex	male	360	226.6 ± 304.0	0.206	110.2 ± 157.4	0.222	176.1 ± 272.9	0.718	175.0 ± 178.5	0.709
	female	18	135.2 ± 146.9		64.5 ± 85.6		151.9 ± 163.3		135.3 ± 87.9	
Age (years)	< 60	160	193.0 ± 264.2	0.093	104.0 ± 148.8	0.663	167.8 ± 240.7	0.656	161.0 ± 144.3	0.248
	≥ 60	218	243.7 ± 320.9		111.0 ± 159.8		180.4 ± 288.3		182.3 ± 195.3	
Smoking	never	22	154.6 ± 240.6	0.269	47.5 ± 48.5	< 0.001	110.3 ± 146.3	0.269	110.6 ± 69.9	0.001
	smoker	342	227.9 ± 304.5		114.3 ± 160.9		177.6 ± 276.3		178.9 ± 181.2	
Tumor size	< 5 cm	284	239.3 ± 322.1	0.037	112.5 ± 155.0	0.330	183.2 ± 284.9	0.233	176.2 ± 177.4	0.570
	≥ 5 cm	94	176.7 ± 209.3		94.5 ± 155.2		149.8 ± 211.2		164.1 ± 169.9	
Lymph node metastasis	absent	230	225.0 ± 290.3	0.852	97.3 ± 133.1	0.108	176.8 ± 272.4	0.905	172.2 ± 167.4	0.850
	present	148	219.1 ± 313.6		125.6 ± 183.7		173.4 ± 264.6		175.7 ± 188.2	
Stage	I/II	298	229.2 ± 311.3	0.384	111.2 ± 158.3	0.445	183.0 ± 287.8	0.272	173.1 ± 168.9	0.982
	III	80	196.3 ± 247.5		96.3 ± 142.8		145.3 ± 178.9		173.6 ± 199.3	

### Relationship between intratumoral CD103^+^ TIL numbers and E-cadherin expression in pSCC tumor cells

Several studies have demonstrated that the interaction between E-cadherin expressed on epithelial cells and CD103 expressed on lymphocytes plays an important role in the retention of antigen-specific lymphocytes within epithelial tissue [17–19]. Thus, we analyzed and compared E-cadherin expression in tumor cells with respect to the number of intratumoral CD103^+^ TILs. The expression of E-cadherin was higher in patients with pSCC (58.3%) compared to those with pADC (29.7%) in NSCLC cohort (Supplementary Table 1) and the H-score of E-cadherin was higher in patients with pSCC (mean, 92.8) than in those with pADC (mean, 52.1) among NSCLC cohort. In pSCC cohort, the mean H-score of E-cadherin is 130.0.

The number of intratumoral CD103^+^ TILs appeared to be higher in pSCC positive for E-cadherin than pSCC negative for E-cadherin expression, but this result was statistically insignificant (Supplementary Table 1). The numbers of intratumoral CD103^+^ TILs were not significantly correlated with E-cadherin expression in tumor cells in the pSCC cohort (Table 2). However, the ratio of intratumoral/stromal CD103^+^ TILs in pSCC was positively associated with E-cadherin expression in tumors (*P* = 0.021).

**Table 2:** The number of CD103+ TILs and the ratio of intratumoral/stromal CD103+TILs according to E-cadherin expression in pulmonary squamous cell carcinoma cohort

			Intratumoral CD103+ TILs		Stromal CD103+TILs		Intratumoral/stromal CD103+ TIL ratio	
		n	Mean ± SD (number/mm2)	P	Mean ± SD (number/mm2)	P	Mean	P
E-cadherin	Negative	97	228.6 ± 296.1	0.787	151.7 ± 198.3	0.001	3.2	0.021
expression	Positive	250	218.7 ± 307.5		80.7 ± 121.2		4.9	

Abbreviations: TILs, tumor infiltrating lymphocytes; SD, standard deviation.

### Prognostic significance of CD103^+^ and CD8^+^ TILs in pSCCs

**Figure 3:**
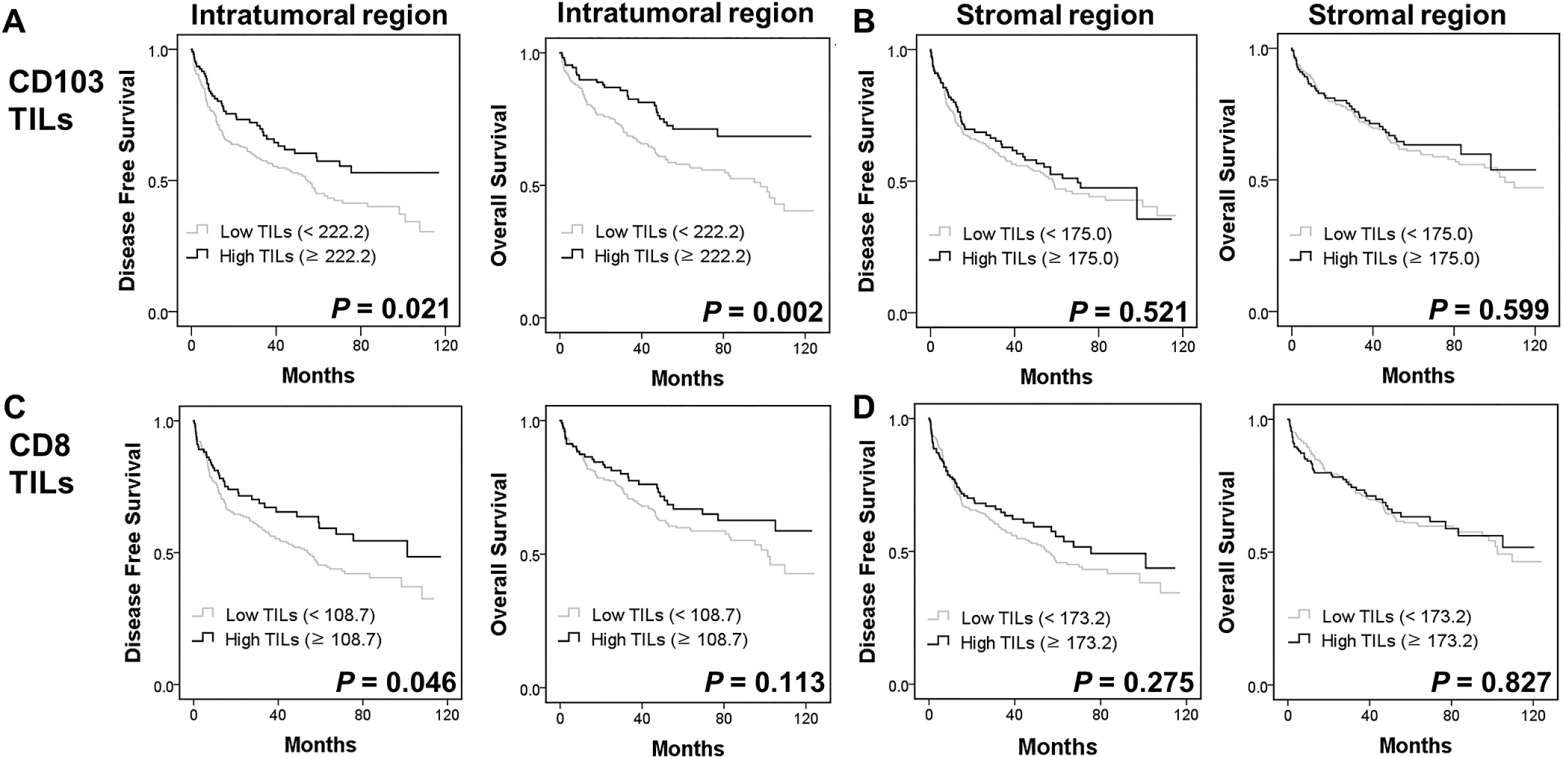
Kaplan-Meier plots using the log rank test for disease-free survival (DFS) and overall survival (OS) in patients with pulmonary squamous cell carcinoma (*n* = 378) according to intratumoral and stromal CD103^+^ (A, B), and CD8^+^ TIL numbers (C, D)

On univariate survival analysis, age, tumor size, tumor stage, and intratumoral CD103^+^ TIL numbers were associated with DFS in patients with pSCC (data not shown). Higher numbers of intratumoral CD103^+^ TILs were significantly associated with prolonged DFS and OS (*P* = 0.021 and 0.002, respectively; Figure 3A), whereas the numbers of intratumoral CD8^+^ TILs was associated with prolonged DFS (*P* = 0.046, Figure 3C) but not OS (*P* = 0.113). The number of stromal CD103^+^ or CD8^+^ TILs was not associated with DFS (*P* = 0.521 and 0.275, respectively) or OS (*P* = 0.599 and 0.827, respectively; Figure 3B and 3D). Furthermore, multivariate analysis revealed that age, lymph node metastasis, and intratumoral CD103^+^ TILs were independent prognostic factors for DFS in pSCC (Table 3).

**Table 3:** Multivariate analysis of disease-free survival outcomes in pulmonary squamous cell carcinoma cohort

	Odds ratio	95% confidence interval	*P* value
Disease-free survival			
Age	2.223	1.576–3.138	< 0.001
Lymph node metastasis	1.551	1.122–2.144	0.008
Intratumoral CD103^+^ TILs	0.648	0.449–0.935	0.021
Stromal CD103^+^ TILs	1.040	0.678–1.593	0.859
Intratumoral CD8^+^ TILs	0.953	0.643–1.413	0.810
Stromal CD8^+^ TILs	0.867	0.542–1.386	0.551

Abbreviations: TILs, tumor-infiltrating lymphocytes.

## DISCUSSION

CD103 is expressed in various immune cells including CD8^+^, CD4^+^ T cells, natural killer cells, natural killer-like T cells, as well as mast cells and dendritic cells [18, 21, 24]. Furthermore, CD8^+^CD103^+^ TILs showed features characteristic of tissue-resident memory T cells that exhibit tumor-specific cytolytic abilities upon stimulation [20]. These findings suggest that CD103^+^ TILs are critically involved in tumor surveillance. Our study demonstrated that a high number of intratumoral CD103^+^ cells was associated with a favorable prognosis in patients with pSCC but not pADC. Consistent with our results, CD103 expression was observed in 10–30% of CD8^+^ TILs isolated from NSCLC tissues [20]. Moreover, high numbers of CD103^+^ cells among intratumoral CD8^+^ T cells have been associated with more favorable prognoses in patients with ovarian and bladder cancers [18, 22]. Based on these results and those from our study, we suggest that the infiltration of CD8^+^CD103^+^ TILs is also a predictor of very favorable prognoses in patients with pSCC, as well as those with ovarian and bladder cancers. Crosstalk between cells in intratumoral and stromal regions might be critical for tumor surveillance, suggesting that investigating CD103 expression patterns in these two tumor regions may enable a more comprehensive understanding of the tumor microenvironment. In this study, the preferential expression of CD103 and CD8 in the immune cells of intratumoral rather than stromal regions was associated with a good prognosis in patients with pSCC. Previously, we reported that CD8^+^ TILs were associated with the expression of PD-1 pathway-related molecules and a better prognosis in patients with pSCC [25], similar to other types of cancers [9–12]. Nevertheless, multivariate survival analysis revealed that the number of intratumoral CD103^+^ TILs is a more powerful predictor of patient survival than the number of CD8^+^ TILs in pSCC.

Meanwhile, tumor-infiltrating regulatory T cell (Treg) also expresses CD103 [26, 27]. To address this, we evaluated the intratumoral and stromal Tregs using immunohistochemistry for FoxP3 (a representative marker of Treg) in pSCC cohort. The numbers of FoxP3^+^ TILs in intratumoral and stromal regions were 18.4 ± 31.6 and 37.7 ± 44.6, respectively (data not shown). The quantity and distribution pattern of Tregs were different from those of CD103^+^ TILs (intratumoral, 222.2 ± 299.0; stromal, 108.7 ± 185.0) and CD8^+^ TILs (intratumoral, 175.0 ± 268.8; stromal, 173.2 ± 175.4). Accordingly, the correlation coefficient between intratumoral CD103^+^ TILs and FoxP3^+^ TILs was much lower than that between intratumoral CD103^+^ TILs and CD8^+^ TILs (0.174 and 0.736, respectively). Consistent with our data, a small proportion of Tregs co-expressed CD103 [27] and the percentage of CD4^+^CD103^+^ TILs was much lower than CD8^+^CD103^+^ TILs in patients with ovarian and bladder cancers [18, 22]. The number of intratumoral and stromal FoxP3^+^ TILs was not significantly associated with DFS and OS of patients with pSCC in this study (data not shown). Based on those previous studies and our present study, it is suggested that the favorable prognostic implication of high intratumoral CD103^+^ TILs may be attributable to the CD8^+^CD103^+^ TILs rather than CD103^+^CD4^+^ Tregs.

In this study, a significant prognostic implication of CD103 expression in TILs was observed in SCC but not in ADC subtype of NSCLC. A high number of intratumoral CD103^+^ TILs was related to improved survival in pSCC. In several studies, the interaction between CD103 and E-cadherin increased granule polarization and exocytosis in TILs [13, 14] and enhanced recruitment and retention of tumor-antigen specific TILs, in ovarian serous carcinoma, glioma, and NSCLC [17–19]. In our study, the H-score and positivity of E-cadherin expression were much higher in pSCC than in pADC. Moreover, a significantly higher ratio of intratumoral/stromal CD103^+^ TILs was observed in pSCC expressing high levels compared to low levels of E-cadherin. Based on these findings, it is feasible that the interaction between CD103 expressed in TILs and E-cadherin expressed in tumor cells might affect tumor immune surveillance and clinical outcomes in patients with pSCC.

Recently, PD-1/PD-L1-targeted immunotherapy was approved by the U.S. Food Drug Administration for the treatment of patients with advanced NSCLC and was deemed a highly promising and safe therapy for cancer [7]. PD-1 expression is induced in T cells that are chronically stimulated by antigens, including viral and tumor antigens [7]. Thus, it is feasible that CD103^+^ T cells entrapped in the intratumoral (i.e., intraepithelial) compartment are exhausted by chronic tumor antigen stimulation, thereby up-regulating PD-1 [28]. Consistent with this suggestion, we observed a strong positive correlation between the number of PD-1^+^ and CD103^+^ TILs in pSCC (*r* = 0.449, *P* < 0.001, data not shown). Moreover, CD8^+^CD103^+^ TILs display a tumor-specific, tissue-resident memory T cell phenotype and frequently express immune checkpoint molecules including PD-1 and Tim-3 [20]. Taken together, we suggest that intratumoral CD103^+^ TILs may serve as a predictive biomarker for PD-1/PD-L1-targeted immunotherapy in NSCLC, as previously proposed [28].

The favorable prognostic implication of high CD103^+^ TILs and the positive correlation between CD103^+^ and PD-1^+^ TILs might seem to be conflicting, considering that PD-1 is a maker of exhausted T cells. Our and other groups demonstrated that a high number of PD-1 TILs was associated with better prognosis in patients with pADC, pSCC and head and neck SCC [12, 25, 29]. It was suggested that increased PD-1^+^ TILs may reflect a preexisting immune response against tumor cells, thus being associated with favorable prognosis of patients with tumor.

Meanwhile, the so-called “immunoscore” has been established as a prognostic biomarker for anti-tumor immune responses [30]. This has been confirmed by many studies using various methods [12, 25, 31, 32]. Among these methods, IHC has emerged as a powerful technology to predict tumor progression and patient survival. However, this method needs to be improved, because interpretation of IHC results is often subjective and dependent on the interpreter. To overcome this limitation, we interpreted IHC results by computer program-based image analysis, which often provides an objective and reproducible interpretation, although standard guidelines need to be established further.

In conclusion, this study suggests that intratumoral CD103^+^ TILs represent an important prognostic biomarker for predicting better survival in patients with pSCC, and as such, may be a potential target candidate for the development of cancer immunotherapies.

## MATERIALS AND METHODS

### Patients and samples

In this study, we established two cohorts: patients with NSCLC and those with pSCC specifically. For the NSCLC cohort, 132 consecutive patients with pADC (*n* = 85) and pSCC (*n* = 47), who underwent resection at Seoul National University Hospital (SNUH), were retrospectively evaluated for the potential prognostic value of CD103^+^ TILs in NSCLC. For the pSCC cohort, 378 patients with pSCC who underwent surgery and had been followed up at SNUH were evaluated to validate the findings in the NSCLC cohort. We excluded those patients who had received chemotherapy before surgery or those who had distant metastasis at the time of diagnosis. Clinicopathologic data and the pathologic tumor-node-metastasis stage according to the 7th American Joint Committee on Cancer were obtained from medical and pathological records. A tissue microarray was constructed from 2 mm diameter cores derived from representative tumor regions of formalin-fixed paraffin-embedded tissue blocks. This study was performed in accordance with the recommendations of the World Medical Association Declaration of Helsinki and was approved by the Institutional Review Board of SNUH (H-1404-100-572).

### Immunohistochemistry

IHC for CD103 was performed using a rabbit monoclonal CD103 antibody (Abcam; Cambridge, UK) and the Benchmark XT autostainer (Ventana Medical Systems; Tucson, AZ, USA). IHC for CD8 (rabbit IgG, clone SP16, Thermo Scientific Fischer; Rockford, IL, USA), Foxp3 (Ab20034, Abcam; Cambridge, UK) and E-cadherin (clone 36B5, Novocastra; New Castle, UK) was performed using the Bond-Max automated immunostainer (Leica Microsystems; Melbourne, Australia). E-cadherin IHC results were evaluated based on staining intensity in tumor cell membranes and scored as follows: : 0, negative; 1, weak or moderate in ≤ 5% of tumor cells; 2, moderate in ≥ 5% of tumor cells; 3, strong in ≥ 5% of tumor cells. A score of 2 or 3 was considered positive for E-cadherin expression. H-score was calculated using the formula, the representative staining intensity of each case × the percentage of expressed tumor cells.

### Automated quantification of CD103^+^, CD8^+^ and FoxP3^+^ TILs

Automated counting of TILs on IHC slides was performed as previously reported.[12, 25] Briefly, slides stained for CD8, CD103 and FoxP3 were scanned using the Aperio ScanScope (Aperio Technologies; Vista, CA, USA), and automated counting was performed using modified nuclear algorithms for IHC in the Aperio ImageScope software (Aperio Technologies). The numbers of intratumoral and stromal CD103^+^, CD8^+^ and FoxP3^+^ TILs per unit area (mm^2^) were calculated and used for statistical analyses.

### Statistical analysis

Quantitative data with a normal distribution are presented as means ± standard deviation. All of the statistical analyses were performed using SPSS software (version 23; IBM Corp., New York, NY, USA). Comparisons between variables were performed using the Student's *t-test*. Cut-off intratumoral or stromal CD103^+^, CD8^+^ and FoxP3^+^ TIL numbers were determined based on the means for categorical analyses. DFS was measured from the date of surgery to that of recurrent or metastatic disease occurrence. OS was measured from the date of diagnosis to that of death from any cause. Survival analysis was performed using the Kaplan-Meier method with the log-rank test. Multivariate Cox regression analysis was performed with consideration of co-linearity. Two-sided *P* values < 0.05 were considered statistically significant.

## SUPPLEMENTARY MATERIALS TABLE AND FIGURE



## References

[1] Lindeman NI, Cagle PT, Beasley MB, Chitale DA, Dacic S, Giaccone G, Jenkins RB, Kwiatkowski DJ, Saldivar JS, Squire J, Thunnissen E, Ladanyi M (2013). College of American Pathologists International Association for the Study of Lung C and Association for Molecular P. Molecular testing guideline for selection of lung cancer patients for EGFR and ALK tyrosine kinase inhibitors: guideline from the College of American Pathologists, International Association for the Study of Lung Cancer, and Association for Molecular Pathology. J Mol Diagn.

[2] Steuer CE, Ramalingam SS (2014). ALK-positive non-small cell lung cancer: mechanisms of resistance and emerging treatment options. Cancer.

[3] Ohashi K, Maruvka YE, Michor F, Pao W (2013). Epidermal growth factor receptor tyrosine kinase inhibitor-resistant disease. J Clin Oncol.

[4] Kim S, Kim TM, Kim DW, Go H, Keam B, Lee SH, Ku JL, Chung DH, Heo DS (2013). Heterogeneity of genetic changes associated with acquired crizotinib resistance in ALK-rearranged lung cancer. J Thorac Oncol.

[5] Albini A, Sporn MB (2007). The tumour microenvironment as a target for chemoprevention. Nat Rev Cancer.

[6] Kerkar SP, Restifo NP (2012). Cellular constituents of immune escape within the tumor microenvironment. Cancer Res.

[7] Topalian SL, Drake CG, Pardoll DM (2015). Immune checkpoint blockade: a common denominator approach to cancer therapy. Cancer Cell.

[8] Apetoh L, Smyth MJ, Drake CG, Abastado JP, Apte RN, Ayyoub M, Blay JY, Bonneville M, Butterfield LH, Caignard A, Castelli C, Cavallo F, Celis E (2015). Consensus nomenclature for CD8 T cell phenotypes in cancer. Oncoimmunology.

[9] Fu J, Xu D, Liu Z, Shi M, Zhao P, Fu B, Zhang Z, Yang H, Zhang H, Zhou C, Yao J, Jin L, Wang H (2007). Increased regulatory T cells correlate with CD8 T-cell impairment and poor survival in hepatocellular carcinoma patients. Gastroenterology.

[10] Sato E, Olson SH, Ahn J, Bundy B, Nishikawa H, Qian F, Jungbluth AA, Frosina D, Gnjatic S, Ambrosone C, Kepner J, Odunsi T, Ritter G (2005). Intraepithelial CD8+ tumor-infiltrating lymphocytes and a high CD8+/regulatory T cell ratio are associated with favorable prognosis in ovarian cancer. Proc Natl Acad Sci USA.

[11] Sharma P, Shen Y, Wen S, Yamada S, Jungbluth AA, Gnjatic S, Bajorin DF, Reuter VE, Herr H, Old LJ, Sato E (2007). CD8 tumor-infiltrating lymphocytes are predictive of survival in muscle-invasive urothelial carcinoma. Proc Natl Acad Sci USA.

[12] Koh J, Go H, Keam B, Kim MY, Nam SJ, Kim TM, Lee SH, Min HS, Kim YT, Kim DW, Jeon YK, Chung DH (2015). Clinicopathologic analysis of programmed cell death-1 and programmed cell death-ligand 1 and 2 expressions in pulmonary adenocarcinoma: comparison with histology and driver oncogenic alteration status. Mod Pathol.

[13] Franciszkiewicz K, Le_Floc'h A, Boutet M, Vergnon I, Schmitt A, Mami-Chouaib F (2013). CD103 or LFA-1 engagement at the immune synapse between cytotoxic T cells and tumor cells promotes maturation and regulates T-cell effector functions. Cancer Res.

[14] Le_Floc'h A, Jalil A, Vergnon I, Le_Maux Chansac B, Lazar V, Bismuth G, Chouaib S, Mami-Chouaib F (2007). Alpha E beta 7 integrin interaction with E-cadherin promotes antitumor CTL activity by triggering lytic granule polarization and exocytosis. J Exp Med.

[15] Agace WW, Higgins JM, Sadasivan B, Brenner MB, Parker CM (2000). T-lymphocyte-epithelial-cell interactions: integrin alpha(E)(CD103)beta, LEEP-CAM and chemokines. Curr Opin Cell Biol.

[16] Mueller SN, Gebhardt T, Carbone FR, Heath WR (2013). Memory T cell subsets, migration patterns, and tissue residence. Annu Rev Immunol.

[17] Jouanneau E, Black KL, Veiga L, Cordner R, Goverdhana S, Zhai Y, Zhang XX, Panwar A, Mardiros A, Wang H, Gragg A, Zandian M, Irvin DK (2014). Intrinsically de-sialylated CD103(+) CD8 T cells mediate beneficial anti-glioma immune responses. Cancer Immunol Immunother.

[18] Webb JR, Milne K, Watson P, Deleeuw RJ, Nelson BH (2014). Tumor-infiltrating lymphocytes expressing the tissue resident memory marker CD103 are associated with increased survival in high-grade serous ovarian cancer. Clin Cancer Res.

[19] Zikos TA, Donnenberg AD, Landreneau RJ, Luketich JD, Donnenberg VS (2011). Lung T-cell subset composition at the time of surgical resection is a prognostic indicator in non-small cell lung cancer. Cancer Immunol Immunother.

[20] Djenidi F, Adam J, Goubar A, Durgeau A, Meurice G, de_Montpreville V, Validire P, Besse B, Mami-Chouaib F (2015). tumor-infiltrating lymphocytes are tumor-specific tissue-resident memory T cells and a prognostic factor for survival in lung cancer patients. J Immunol.

[21] Quinn E, Hawkins N, Yip YL, Suter C, Ward R (2003). CD103+ intraepithelial lymphocytes—a unique population in microsatellite unstable sporadic colorectal cancer. Eur J Cancer.

[22] Wang B, Wu S, Zeng H, Liu Z, Dong W, He W, Chen X, Dong X, Zheng L, Lin T, Huang J (2015). Tumor Infiltrating Lymphocytes Predict a Favorable Prognosis in Urothelial Cell Carcinoma of the Bladder. J Urol.

[23] Zhang N, Bevan MJ (2013). Transforming growth factor-beta signaling controls the formation and maintenance of gut-resident memory T cells by regulating migration and retention. Immunity.

[24] Sung SS, Fu SM, Rose CE, Gaskin F, Ju ST, Beaty SR (2006). A major lung CD103 (alphaE)-beta7 integrin-positive epithelial dendritic cell population expressing Langerin and tight junction proteins. J Immunol.

[25] Kim MY, Koh J, Kim S, Go H, Jeon YK, Chung DH (2015). Clinicopathological analysis of PD-L1 and PD-L2 expression in pulmonary squamous cell carcinoma: Comparison with tumor-infiltrating T cells and the status of oncogenic drivers. Lung Cancer.

[26] Anz D, Mueller W, Golic M, Kunz WG, Rapp M, Koelzer VH, Ellermeier J, Ellwart JW, Schnurr M, Bourquin C, Endres S (2011). CD103 is a hallmark of tumor-infiltrating regulatory T cells. Int J Cancer.

[27] Allakhverdi Z, Fitzpatrick D, Boisvert A, Baba N, Bouguermouh S, Sarfati M, Delespesse G (2006). Expression of CD103 identifies human regulatory T-cell subsets. J Allergy Clin Immunol.

[28] Webb JR, Milne K, Nelson BH (2014). Location, location, location: CD103 demarcates intraepithelial, prognostically favorable CD8 tumor-infiltrating lymphocytes in ovarian cancer. Oncoimmunology.

[29] Badoual C, Hans S, Merillon N, Van Ryswick C, Ravel P, Benhamouda N, Levionnois E, Nizard M, Si-Mohamed A, Besnier N, Gey A, Rotem-Yehudar R, Pere H, T (2013). PD-1-expressing tumor-infiltrating T cells are a favorable prognostic biomarker in HPV-associated head and neck cancer. Cancer Res.

[30] Angell H, Galon J (2013). From the immune contexture to the Immunoscore: the role of prognostic and predictive immune markers in cancer. Curr Opin Immunol.

[31] Liu S, Lachapelle J, Leung S, Gao D, Foulkes WD, Nielsen TO (2012). CD8+ lymphocyte infiltration is an independent favorable prognostic indicator in basal-like breast cancer. Breast Cancer Res.

[32] Mahmoud SM, Paish EC, Powe DG, Macmillan RD, Grainge MJ, Lee AH, Ellis IO, Green AR (2011). Tumor-infiltrating CD8+ lymphocytes predict clinical outcome in breast cancer. J Clin Oncol.

