# Supplementary material for: Prognostic implications of intratumoral CD103+ tumor-infiltrating lymphocytes in pulmonary squamous cell carcinoma
Source: Oncotarget. 2017 Jan 10;8(8):13762–9. doi: 10.18632/oncotarget.14632 (PMC5355136; doi:10.18632/oncotarget.14632)
Supplement: Supplementary file 1 [file oncotarget-08-13762-s001.pdf]

# Prognostic implications of intratumoral CD103+ tumor-infiltrating lymphocytes in pulmonary squamous cell carcinoma

## Supplementary Materials

**Supplementary Table 1: The numbers of intratumoral CD103$^+$ tumor-infiltrating lymphocytes (TILs) according to patients clinicopathological characteristics in non-small cell lung cancer cohort**

| | | SCC | Intratumoral CD103$^+$ TILs | | ADC | Intratumoral CD103$^+$ TILs | |
|---|---|---|---|---|---|---|---|
| Clinicopathological characteristics | | *n* | Mean ± SD (number/mm$^2$) | *P* | *n* | Mean ± SD (number/mm$^2$) | *P* |
| Sex | male | 46 | 360.8 ± 372.2 | 0.683 | 38 | 237.7 ± 271.2 | 0.051 |
| | female | 1 | 222.2 | | 47 | 138.6 ± 159.4 | |
| Age (years) | < 60 | 12 | 302.9 ± 314.1 | 0.511 | 39 | 200.4 ± 285.0 | 0.525 |
| | ≥ 60 | 35 | 385.4 ± 389.1 | | 46 | 168.1 ± 148.3 | |
| Smoking | never | 2 | 238.8 ± 238.6 | 0.629 | 52 | 134.0 ± 155.8 | 0.023 |
| | smoker | 45 | 369.9 ± 375.5 | | 33 | 259.9 ± 281.8 | |
| Tumor size | < 5 cm | 14 | 387.8 ± 387.2 | 0.304 | 80 | 178.2 ± 208.3 | 0.683 |
| | ≥ 5 cm | 33 | 230.5 ± 222.6 | | 5 | 257.4 ± 400.5 | |
| Lymph node metastasis | absent | 31 | 359.7 ± 307.8 | 0.906 | 61 | 196.7 ± 242.9 | 0.362 |
| | present | 16 | 373.4 ± 479.5 | | 24 | 147.8 ± 149.6 | |
| Stage | I/II | 41 | 345.0 ± 332.8 | 0.539 | 68 | 195.4 ± 233.0 | 0.298 |
| | III | 6 | 445.4 ± 600.3 | | 17 | 132.7 ± 159.4 | |
| Event[a] | absent | 21 | 468.1 ± 419.5 | 0.055 | 54 | 184.7 ± 231.8 | 0.920 |
| | present | 26 | 264.0 ± 297.0 | | 31 | 179.7 ± 204.1 | |
| E-cadherin expression | negative | 15 | 364.9 ± 402.2 | 0.562 | 52 | 195.9 ± 218.4 | 0.149 |
| | positive | 21 | 445.0 ± 406.9 | | 22 | 117.3 ± 195.5 | |

[a]Presence of recurrence or death.
Abbreviations: SCC, squamous cell carcinoma; ADC, adenocarcinoma.

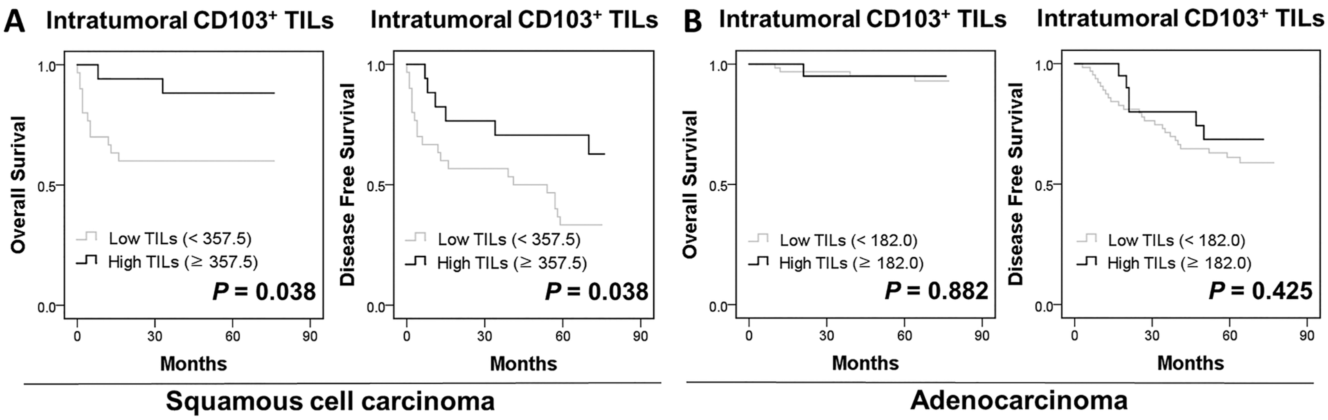


**Supplementary Figure 1:** Kaplan-Meier plots using the log rank test for overall survival (OS) and disease-free survival (DFS) in the non-small cell lung cancer cohort including patients with pulmonary squamous cell carcinoma (pSCC) ($n$ = 47) (**A**) and pulmonary adenocarcinoma (pADC) ($n$ = 85) (**B**) according to intratumoral CD103$^+$ TIL numbers.
